# Supplementary material for: Cyto- and bio-compatibility assessment of plasma-treated polyvinylidene fluoride scaffolds for cardiac tissue engineering
Source: Front Bioeng Biotechnol. 2022 Nov 4;10:1008436. doi: 10.3389/fbioe.2022.1008436 (PMC9672675; doi:10.3389/fbioe.2022.1008436)
Supplement: Supplementary file 1 [file DataSheet1.docx]

Supplementary Material

Cyto- and bio-compatibility assessment of plasma-treated polyvinylidene fluoride (PVDF) scaffolds for cardiac tissue engineering

**Maria Kitsara^1*^, Gaëlle Revet^1^, Alexandre Simon^1^, Mathilde Minguy^1^, Antoine Miche^3^, Vincent Humblot^3,4^, Thierry Dufour^2*^, Onnik Agbulut^1*^**

^1^Biological Adaptation and Ageing, UMR CNRS 8256, INSERM ERL 1164, Institut de Biologie Paris-Seine, Sorbonne Université, Paris, 75005, France

^2^Laboratoire de Physique des Plasmas, UMR CNRS 7648, Sorbonne Université, Paris, 75005, France

^3^Laboratoire de Réactivité de Surface, UMR CNRS 7197, Sorbonne Université, Paris, 75005, France

^3^FEMTO-ST Institute, UMR 6174 CNRS, Université Bourgogne Franche-Comté, 15B avenue Montboucons, 23030 Besançon Cedex, France

*** Correspondence:**Dr Maria Kitsara : [kitsara.m@gmail.com](mailto:kitsara.m@gmail.com)

Dr Thierry Dufour : [thierry.dufour@sorbonne-universite.fr](mailto:thierry.dufour@sorbonne-universite.fr)

Prof Onnik Agbulut : [onnik.agbulut@sorbonne-universite.fr](mailto:onnik.agbulut@sorbonne-universite.fr)

**Table S1. Primers sequences used in this study.**

| **Gene** | **Forward (5'-3')** | **Reverse (5'-3')** |
| --- | --- | --- |
| *Actn2* | AGACATGGGCTTACGGCAAA | GCTGCAATCTGTTCCACACG |
| *Myh7* | CAACCTGTCCAAGTTCCGC | TACTCTTCATTCAGGCCCTTGG |
| *Pln* | GCTCCCAGACTTCACACAAC | TCTCCTTTTAGGAGGCCTTGG |
| *Atp2a2* | TCTGCTTGTCCATGTCCCTT | ATTGCAGGCTCCAGGTAGTT |
| *Cryab* | GGAGAGCACCTGTTGGAGTC | CATACGCATCTCTGAGAGCCC |
| *Cdh2* | CACCCGGCTTAAGGGTGATT | CGATCCTGTCTACGTCGGTG |
| *Cdh13* | TGGTCAAGCCCCTGGACTAT | ACCATCATGGGGTCTGGGTA |
| *Col1a1* | TGTGCCTCAGAAGAACTGGT | CGCTTCCATACTCGAACTGG |
| *ECM1* | ATAAAGACCCACCCCCACTC | TCCACAGAGATGGTCCATGA |
| *ELN* | GCTAAATACGGAGCAGCAGG | TACTCCACCAGGAACACCAC |
| *Lama4* | TCACCACACCGATGGCTAAC | TGAGGTTTCTCACTGCGTCC |
| *Lamb2* | GTCTTCGCTGTGACCACTGT | AACCAGCAATGCACCTCTCA |
| *Lamc1* | TTCTACAACCTGCAGAGCGG | TGGTGACAGTCGCAAGGTTT |
| *Actb* | AGATCAAGATCATTGCTCCTCCT | AAGGGTGTAAAACGCAGCTC |
| *B2m* | TGAATTCACACCCACCGAGA | TACATGTCTCGGTCCCAGGT |
| *Nubp1* | CCCAAGTGCAAGAGAGAGTC | ACTTTGCCCAGAAGAGGGAT |

**Table S2. Fold changes of mRNA expression of the genes used to construct the cardiac and the adhesion index.** Non-treated surface values are set at 1.0. Values are given as the means ± standard error of the mean (SEM).

| **Gene** | **Index** | **Ar** | **Ar-N_2_** | **He** | **He-N_2_** |
| --- | --- | --- | --- | --- | --- |
| *Actn2* | Cardiac index | 1.30 ± 0.53 | 3.63 ± 1.90 | 1.65 ± 1.31 | 0.98 ± 0.50 |
| *Atp2a2* |  | 1.10 ± 0.40 | 5.08 ± 2.59 | 1.43 ± 1.10 | 1.09 ± 0.53 |
| *Cryab* |  | 0.73 ± 0.27 | 1.61 ± 0.85 | 1.14 ± 0.44 | 1.46 ± 0.52 |
| *Myh7* |  | 1.78 ± 0.55 | 4.38 ± 2.33 | 2.66 ± 1.28 | 1.79 ± 0.65 |
| *Pln* |  | 1.64 ± 0.49 | 2.32 ± 1.49 | 1.18 ± 0.95 | 1.39 ± 0.87 |
| *Cdh13* | Adhesion index | 0.85 ± 0.47 | 1.78 ± 0.36 | 0.75 ± 0.40 | 1.00 ± 0.35 |
| *Cdh2* |  | 2.65 ± 0.82 | 2.78 ± 0.81 | 1.98 ± 0.84 | 2.78 ± 0.61 |
| *Col1a1* |  | 1.78 ± 0.71 | 1.79 ± 1.10 | 1.35 ± 0.64 | 1.72 ± 0.53 |
| *Ecm1* |  | 1.63 ± 0.44 | 1.61 ± 0.76 | 1.65 ± 0.94 | 1.43 ± 0.34 |
| *Eln* |  | 1.56 ± 0.47 | 1.32 ± 1.06 | 0.75 ± 0.37 | 1.23 ± 0.63 |
| *Lama4* |  | 1.94 ± 0.66 | 1.70 ± 0.19 | 2.27 ± 0.95 | 1.53 ± 0.11 |
| *Lamb2* |  | 1.27 ± 0.34 | 2.05 ± 1.27 | 1.19 ± 0.73 | 1.71 ± 1.00 |
| *Lamc1* |  | 1.39 ± 0.15 | 1.68 ± 0.57 | 1.52 ± 0.42 | 1.98 ± 0.26 |

**Table S3. Fold changes of mRNA expression of the genes used to construct the cardiac and the adhesion index before normalization.** Values are given as the means ± standard error of the mean (SEM).

| **Gene** | **Index** | **Non-treated** | **Ar** | **Ar-N_2_** | **He** | **He-N_2_** |
| --- | --- | --- | --- | --- | --- | --- |
| *Actn2* | Cardiac index | 4.33 ± 0.53 | 5.63 ± 2.28 | 15.72 ± 8.24 | 7.16 ± 5.68 | 4.23 + 2.16 |
| *Atp2a2* |  | 10.03 ± 1.54 | 11.05 ± 3.97 | 50.99 ± 26.01 | 14.37 ± 11.09 | 10.99 ± 5.36 |
| *Cryab* |  | 5.98 ± 0.85 | 4.37 ± 1.64 | 9.61 ± 5.09 | 6.79 ± 2.64 | 8.76 ± 3.10 |
| *Myh7* |  | 1.22 ± 0.15 | 2.18 ± 0.67 | 5.35 ± 2.84 | 3.24 ± 1.56 | 2.19 ± 0.80 |
| *Pln* |  | 1.18 ± 1.03 | 1.93 ± 0.58 | 2.73 ± 1.76 | 1.39 ± 1.12 | 1.64 ±1.03 |
| *Cdh13* | Adhesion index | 0.57 ± 0.01 | 0.48 ± 0.27 | 1.01 ± 0.20 | 0.43 ± 0.23 | 0.57 ± 0.20 |
| *Cdh2* |  | 0.33 ± 0.10 | 0.87 ± 0.27 | 0.92 ± 0.27 | 0.65 ± 0.28 | 0.92 ± 0.20 |
| *Col1a1* |  | 13.71 ± 1.48 | 24.44 ± 9.72 | 24.51 ± 15.01 | 18.47 ± 8,74 | 23.54 ± 7.28 |
| *Ecm1* |  | 0.03 ± 0.01 | 0.05 ± 0.01 | 0.05 ± 0.03 | 0.06 ± 0.03 | 0.05 ± 0.01 |
| *Eln* |  | 1.24 ± 0.25 | 1.92 ± 0.58 | 1.63 ± 1.31 | 0.93 ± 0.46 | 1.51 ± 0.78 |
| *Lama4* |  | 0.08 ± 0.02 | 0.16 ± 0.06 | 0.14 ± 0.02 | 0.19 ± 0.08 | 0.13 ± 0.01 |
| *Lamb2* |  | 0.28 ± 0.07 | 0.36 ± 0.10 | 0.58 ± 0.36 | 0.34 ± 0.21 | 0.48 ± 0.28 |
| *Lamc1* |  | 0.67 ± 0.11 | 0.93 ± 0.10 | 1.13 ± 0.39 | 1.02 ± 0.28 | 1.33 ± 0.17 |

**Supplementary Figure S1.** **Scanning electron microscope (SEM) images of drop-cast scaffolds:** (a) Native PVDF scaffold obtained by 5% w/v solution, (b) PVDF scaffold after Ar plasma exposure, (c) PVDF scaffold after He plasma exposure, (d) PVDF scaffold after Ar-N_2_ plasma exposure, (e) PVDF scaffold after He-N_2_ plasma exposure. The images (b), (c), (d) and (e) correspond to the side exposed to plasma.
